# Molecular characterization of mutations in white-flowered torenia plants

**DOI:** 10.1186/1471-2229-14-86

**Published:** 2014-04-02

**Authors:** Masahiro Nishihara, Eri Yamada, Misa Saito, Kohei Fujita, Hideyuki Takahashi, Takashi Nakatsuka

**Affiliations:** 1 Iwate Biotechnology Research Center, Narita 22-174-4, Kitakami, Iwate 024-0003, Japan; 2 Department of Biological and Environmental Science, Graduate School of Agriculture, Shizuoka University, 836 Ohya Suruga-ku, Shizuoka 422-8529, Japan

**Keywords:** *Torenia fournieri*, *F3H*, Muation, LTR-type retrotransposon, White flower

## Abstract

**Background:**

Torenia (*Torenia fournieri* Lind.) is a model plant increasingly exploited in studies in various disciplines, including plant engineering, biochemistry, physiology, and ecology. Additionally, cultivars with different flower colors have been bred and made commercially available. Flower color in torenia is mainly attributed to the accumulation of anthocyanins, but the molecular mechanisms inducing flower color mutations in torenia have not been well elucidated. In this study, we therefore attempted to identify the cause of white coloration in torenia by comparing the white-flowered cultivar Crown White (CrW) with Crown Violet (CrV), a violet-flowered variety.

**Results:**

In an expression analysis, no flavanone 3-hydroxylase (*TfF3H*) transcript accumulation was detected in CrW petals. Sequence analyses revealed that a novel long terminal repeat (LTR)-type retrotransposable element, designated as *TORE1* (*Torenia* retrotransposon 1), is inserted into the 5*′*-upstream region of the *TfF3H* gene in CrW. A transient expression assay using torenia *F3H* promoters with or without *TORE1* insertion showed that the *TORE1* insertion substantially suppressed *F3H* promoter activity, suggesting that this insertion is responsible for the absence of *F3H* transcripts in white petals. Furthermore, a transformation experiment demonstrated that the introduction of a foreign gentian *F3H* cDNA, *GtF3H*, into CrW was able to recover pink-flower pigmentation, indicating that *F3H* deficiency is indeed the cause of the colorless flower phenotype in CrW. Detailed sequence analysis also identified deletion mutations in flavonoid 3′-hydroxylase (*TfF3′H*) and flavonoid 3′,5′- hydroxylase (*TfF3′5′H*) genes, but these were not directly responsible for white coloration in this cultivar.

**Conclusions:**

Taken together, a novel retrotransposable element, *TORE1,* inserted into the *F3H* 5′-upstream region is the cause of deficient *F3H* transcripts in white-flowered torenia, thereby leading to reduced petal anthocyanin levels. This is the first report of a retrotransposable element involved in flower color mutation in the genus *Torenia*.

## Background

Natural or spontaneous mutations, which accelerate evolution in living organisms, have a number of causes. In flowering plants, flower color mutations occur widely in nature, and floricultural plants in a variety of flower colors have been produced through artificial selection of this natural variability. Flavonoids, such as flavones, aurones, flavonols, and anthocyanins, are the most important plant pigments associated with flower coloration. The flavonoid biosynthetic pathway is one of the most extensively studied pathways in plant specialized metabolism [1]. The biosynthetic genes necessary for biosynthesis of colored anthocyanins have been especially well studied in many plant species, including, Arabidopsis, snapdragon, petunia, grape, and maize, and have become targets for molecular breeding [1-4]. In addition to flower coloration, studies of anthocyanin-based coloration and associated mutations in fruits, seeds, and other organs have provided information helpful for elucidation of flavonoid biosynthesis-related genes. In particular, recent molecular biological investigations have revealed the basic mechanisms generating these mutations and their effects on pigmentation at the molecular level in different plant species.

Torenia (*Torenia fournieri* Lind., also known as wishbone flower) is a perennial plant widely used as a bedding flower from early spring through summer. Cultivars in different flower colors, such as white, blue, and pink, have been generated by conventional breeding and are now commercially available. Because of its various merits in regard to molecular analyses (reviewed in [5,6]), the species is proposed as a potential new model flower to study a wide range of floral traits. *Torenia fournieri* and *T. hybrida* (*T. fournieri* × *T. concolor*) are frequently used in transgenic studies of various subjects such as flower color [7-9], anthocyanin synthesis [10], chlorophyll degradation [10], flower shape [11-14], and fertilization [15]. Among studied characteristics, flower color is one of the most targeted traits in molecular investigations, as flower color modification is of strong interest from both basic and applied research perspectives. Torenia flower pigments are composed of flavonoid anthocyanins. Chemical analysis has revealed the anthocyanin components of *T. fournieri* ‘Crown Violet’ [7] and *T. hybrida* ‘Summerwave Blue’ [16]. Using these cultivars, flower colors ranging from the original violet to white, pink, and yellow have been successfully produced by genetic engineering of flavonoid biosynthetic genes [7-9,16-19].

As mentioned above, variously flower-colored torenia cultivars and lines have been produced by both conventional and molecular methods, but the origins of color mutations in the breeding materials are largely unknown. Because they are easily recognizable by eye and hence good plant research materials, flower color mutations have been studied extensively in species such as petunia [20,21], snapdragon [22,23], and morning glory [24,25]. For example, the variegated-flower morning glory mutation is caused by the insertion of the *Tpn1* transposable element into the dihydroflavonol 4-reductase *(DFR)* gene [26], and white-flowered morning glory is derived from insertions of *Tpn1*-related DNA transposable elements into an intron of the chalcone synthase (*CHS*) gene [27]. Red-flowered phenotypes in commercial petunias have been attributed to transposon insertion mutations of the *F3′5′H* gene [28]. Similarly, various mutations related to flower pigmentation have been studied in carnation [29-31], with class I, class II, and other transposable elements implicated in the color changes. We have also identified several DNA- and RNA-type transposable elements in white- or pink-flowered mutants in Japanese gentian flowers [32-34]. With respect to torenia, little is known regarding the source of flower color mutations, with one exception: Nishijima et al. [35] recently reported that a ‘flecked’ mutant, bearing variegated flowers, originated through the insertion of an *Enhancer/Suppressor-Mutator* (*En/Spm*)-like transposon (*Ttf1*) into the intron of the *TfMYB1* transcription factor gene. The causal agents of flower color mutations in other torenia cultivars have not yet been identified.

In the present study, we uncovered and analyzed mutations in a white-flowered torenia through comparison with a violet-flowered cultivar. We first identified a novel retrotransposable element, designated *TORE1*, in torenia based on a molecular biological approach. Using both transient and stable transformation experiments, we then confirmed that the insertion of *TORE1* into the flavanone 3-hydroxylase (*F3H*) promoter region contributes to the white-flowered phenotype. We found two additional mutations in this cultivar and considered their association with white coloration. This is the first known identification of a retrotransposable element in the genus *Torenia*.

## Results

### Analysis of flavonoid components in torenia flowers

**Figure 1:**
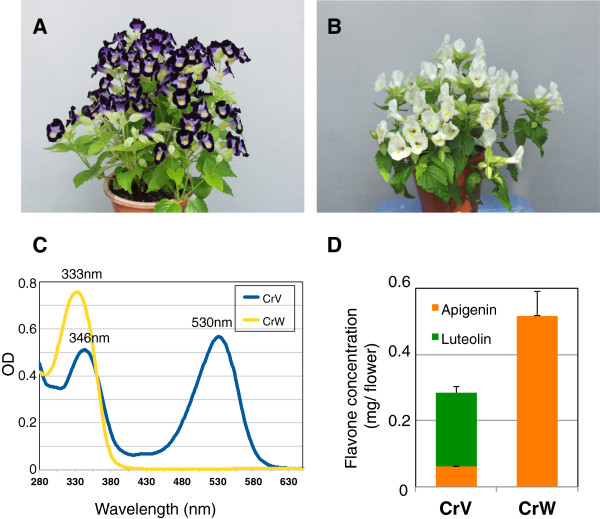
**Torenia cultivars used in this study. (A)** Crown Violet. **(B)** Crown White. **(C)** Absorbance spectrum of 0.1% HCl-methanol extracts of flower petals. **(D)** Flavone aglycone concentrations of CrV and CrW determined by HPLC analysis. Averages of four flowers ± standard deviations are shown.

As shown in Figure 1A and 1B, *T. fournieri* cultivars Crown Violet (CrV) and Crown White (CrW) have violet and white flower petals, respectively. Spectral profiles of 0.1% HCl-methanol extracts of each flower are shown in Figure 1C. In the visible light region, CrV had a maximum absorption wavelength at 530 nm corresponding to anthocyanin pigments; in contrast, CrW had no detectable peak in the visible spectrum, indicating that no anthocyanins accumulated in this white-flowered cultivar. UV absorption spectra revealed that CrW and CrV had maximum absorption peaks at 346 and 333 nm, respectively. To identify the flavone components accumulating in each cultivar, acid hydrolysis was performed followed by HPLC analysis. The flavone aglycones identified in CrV, as described previously [7], were apigenin and luteolin, whereas CrW contained only apigenin. Total levels of flavone derivatives were 1.8 times higher in CrW than in CrV (Figure 1D). These results suggest one possibility that the hydroxylation activity of flavonoid B-rings is deficient in CrW.

### Expression analysis of flavonoid biosynthetic genes in torenia flowers

**Figure 2:**
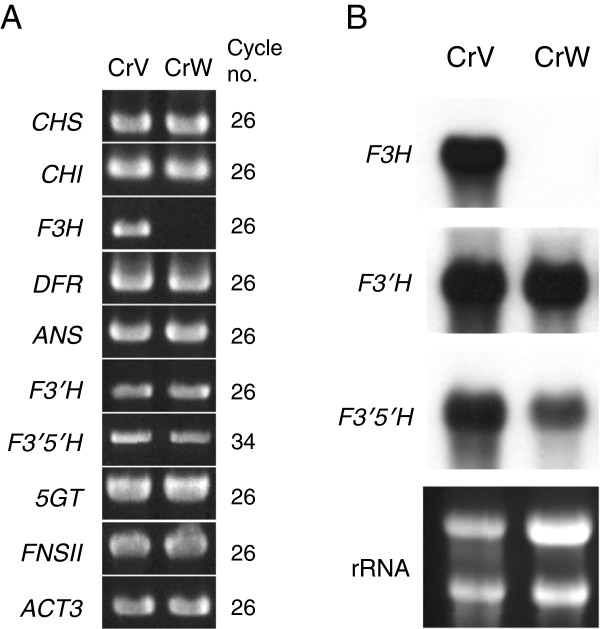
**Expression analyses of flavonoid biosynthetic genes in CrV and CrW. (A)** RT-PCR analysis of nine different flavonoid biosynthetic genes and the β-actin gene (internal control). Gene names and PCR cycles are shown to the left and right of each panel, respectively. **(B)** Northern blot analysis of *F3H*, *F3′H* and *F3′5′H* genes in torenia flowers. *rRNA* was used as a total RNA loading control.

We attempted to screen the mutated genes by means of an expression analysis of torenia flower petals. As shown in Additional file 1: Table S1, we designed primers based on flavonoid biosynthetic genes of *T. hybrida*, because full-length sequences, except for chalcone isomerase *(CHI)*, are available for this hybrid cultivar. Using these primers, which were designed for nearly full-length amplification, RT-PCR analysis was performed on the flavonoid biosynthesis-related genes *CHS*, *CHI*, *F3H*, *DFR*, anthocyanidin synthase *(ANS)*, *F3′H, F3′5′H,* anthocyanin 5-*O*-glucosyltransferase *(5GT),* and flavone synthase II *(FNSII)* (Figure 2A)*.* Fragments of the expected length were amplified from each gene, and similar expressions were observed between the two cultivars for all genes except for *F3H*. Expressions of *TfMYB1* and *TfbHLH1*, recently identified as transcription factor genes regulating anthocyanin biosynthesis in torenia flowers [35], also did not differ significantly between the two cultivars (Additional file 2: Figure S1). Furthermore, attempts to amplify partial fragments of *F3H* using several different primers were unsuccessful (data not shown), indicating that *F3H* expression was completely suppressed in CrW. To elucidate the expressions of *F3H*, *F3′H*, and *F3′5′H,* we next performed northern blot analyses using the same RNAs (Figure 2B). No *F3H* transcripts were detected in the analyses, confirming the RT-PCR results. With respect to the other two hydroxylases, *F3′H* expression remained unchanged and *F3′5′H* expression was slightly decreased. No visible band shifts were detected, indicating that no large indels were present in these two genes.

### Identification of genomic and cDNA sequences of three flavonoid biosynthetic genes in torenia plants

Based on the results of the pigment and expression analyses, we speculated that three genes, *F3H*, *F3′H*, and *F3′5′H,* were causally related to the CrW flower color mutation. We therefore compared genomic sequences of these three genes between the two cultivars. We generated DNA fragments that spanned start and stop codons using genomic PCR, and analyzed the sequences. *F3H* was identical between CrV and CrW*,* whereas deletion mutations were present in *F3′H* and *F3′5′H* genomic sequences in both CrV and CrW (Additional file 2: Figure S2). In particular, a 12-bp deletion was observed in exon 3 of the CrW *F3′H* gene, presumably lacking four amino acid residues (Additional file 2: Figure S2A). The *F3′5′H* gene from CrW also had a single-base (cytosine) deletion in exon 1, resulting in a frame shift (Additional file 2: Figure S2B). These mutated *F3′H* and *F3′5′H* sequences were also present in CrV, indicating that CrV is heterozygous for both gene mutations.

### Isolation of the 5′-upstream region of the *F3H* gene from torenia plants

**Figure 3:**
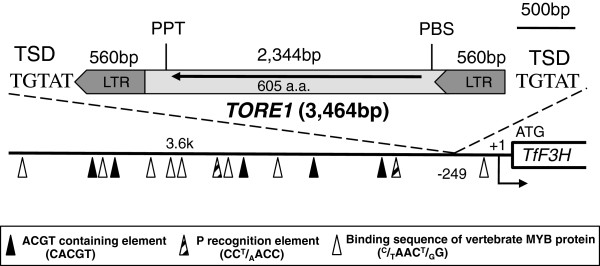
**Schematic diagram of the genomic structure of the *F3H *gene in torenia.** An insertion of *TORE1* within the 5*′*-upstream region of *F3H* was identified in CrW. *TORE1* has the features of long terminal repeat (LTR) retrotransposons, containing 5 bp of the target site duplication (TSD) and 560 bp of LTRs. A deduced open reading frame (ORF) is indicated by the black arrow. The primer binding site (PBS) and polypurine tract (PPT) are also shown.

Because no mutation was found in the *F3H* gene, including coding exon and intron sequences, we suspected that CrW might have a specific mutation in the 5′-upstream region of *F3H*. To clone the *F3H* upstream region, an inverse PCR was therefore performed. A 3,898-bp sequence upstream of the start codon was identified in CrV (accession no. AB902919) and found to contain *cis*-motifs such as a P-recognition element, an ACGT-containing element, and several MYB-related elements (Figure 3). Interestingly, the *F3H* upstream region of CrW (accession no. AB902920) contained a long 3,464-bp insertion at the -249th position prior to the transcription start site (Figure 3). This insertion had the features of an LTR-type retrotransposon, and was accordingly designated as *Torenia* retrotransposon 1 (*TORE1*). Our analysis revealed that *TORE1* contains a completely identical pair of LTRs (560 bp) that start with TG and end with CA—a typical canonical sequence—flanked by 5-bp direct repeats of the *TfF3H* sequence. *TORE1* also contains a single open reading frame (ORF) encoding a putative gag-pol polyprotein of 605 amino acid residues. A blastp search revealed that the protein has partial homology to hypothetical proteins of *Vitis vinifera*, the *Copia-like* LTR Rider of *Solanum lycopersicum*[36], and a putative gag-pol polyprotein of *Citrus sinensis*[37]. The protein contains gag and protease, but seemingly lacks typical pol proteins such as integrase, reverse transcriptase, and RNaseH. As shown in Additional file 2: Figure S3, a primer binding site (PBS) and a polypurine tract (PPT) are also present in *TORE1*. In addition to the complete length of *TORE1*, genomic PCR amplification of CrW yielded a 5′-upstream fragment of *F3H* containing only a solo LTR (Additional file 2: Figure S4). In CrV, the amplified 5′-upstream region of *F3H* within 3.9 kb of the start codon did not contain *TORE1* or *TORE1*-related sequences.

### Southern blot analysis of three flavonoid biosynthetic genes and *TORE1* in torenia

**Figure 4:**
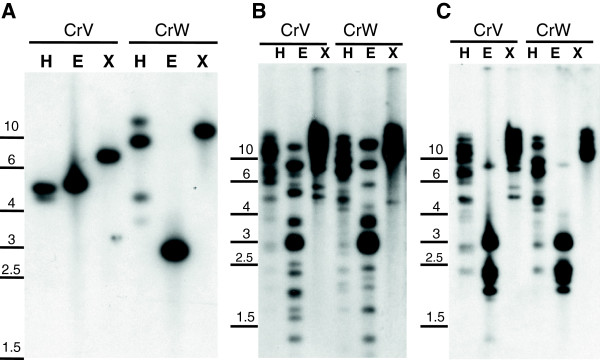
**Southern blot analysis of *F3H *and *TORE1 *in CrV and CrW.** Total genomic DNAs were digested with *Hin*d III (H), *Eco* RI (E), and *Xba* I (X) and transferred to nylon membranes as described in Methods. Membranes were probed with DIG-labeled sequences of *F3H***(A)**, LTR **(B)**, and gag-pol protein **(C)** from *TORE1*. DNA marker sizes (kbp) are shown.

To determine the status of the three mutated genes and the retrotransposable element *TORE1* in each torenia cultivar, Southern blot analyses were performed (Figure 4; Additional file 2: Figure S5). Digestion with *Eco*R I and *Xba* I, which do not cleave the *F3H* gene containing three exons and two introns, confirmed that the *F3H* gene is present as a single copy gene in the torenia genome (Figure 4A). Digestion with *Hin*d III, which cleaves the second *F3H* intron, generated two and four bands from CrV and CrW, respectively. The larger bands generated from CrW may have been due to partial *Hin*d III digestion within *TORE1*, as depicted in Additional file 2: Figure S6. Southern blot analyses, using as probes the 560-bp LTR (Figure 4B) or the gag-pol polyprotein sequence of 605 deduced amino acids (Figure 4C), uncovered several bands from all restriction enzymes, suggesting that *TORE1* or related elements are ubiquitously present in the torenia genome. *F3′H* and *F3′5′H* genes were also found in single copies in the torenia genome (Additional file 2: Figure S5). Additional banding patterns were observed in CrV, reflecting the heterozygosity of CrV mentioned above.

### Transient expression analysis for *F3H* promoter activity

**Figure 5:**
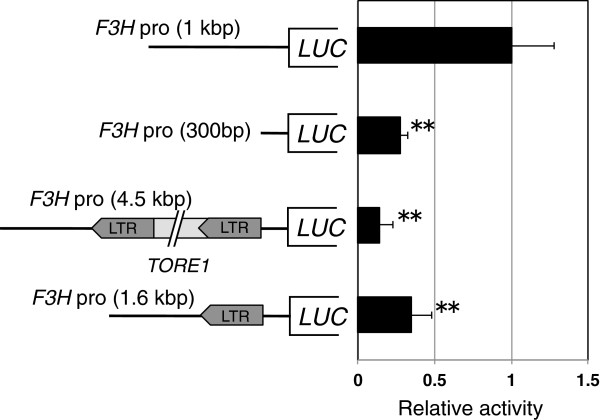
**Transient expression assay for activation ability of *F3H *promoters in Arabidopsis suspension cells.** Suspension-cultured cells of *Arabidopsis thaliana* (T87 line) were used for this analysis. *GtMYB3* and *GtbHLH1* genes that can activate late-stage anthocyanin biosynthetic genes were co-introduced with various *F3H* promoters via PEG-mediated transformation. After 24 h culture, firefly luciferase (FLUC) activity was measured with a luminometer. A *Renilla* luciferase (RLUC)-driven Cauliflower mosaic virus (CaMV) 35S promoter was used for internal standardization of PEG infection. Relative activities (LUC/RLUC) are shown. Asterisks (**) indicate significant differences between *TfF3H* pro (1 kbp) and other constructs (*P* < 0.01, *t*-test).

Because the *TORE1* insertion into the 5′-upstream region most likely has a deleterious effect on *F3H* promoter activity, we measured this activity using a transient expression system in protoplasts of Arabidopsis suspension-cultured cells. Transcription factors GtMYB3 and GtbHLH1, isolated from gentian petals, are known to activate late-stage flavonoid biosynthetic genes [33,38]; we therefore used these two transcription factors as effectors in combination with different torenia *F3H* promoter-firefly luciferase gene (*LUC*) constructs, as shown in Figure 5. The 35Spro- *Renilla* luciferase gene (*RLUC*) construct was also co-transformed as an internal standard. The approximately 1-kbp 5′-upstream region of the torenia *F3H* gene was used for this analysis. This 1-kbp promoter was assigned a LUC/RLUC relative activity of 1. Compared with the 1-kb promoter, the 300-bp *TfF3H* promoter showed only 27.7% activity; the *TORE1*-inserted *TfF3H* promoter displayed an even greater activity reduction, down to 14.3%. Insertion of the solo-LTR sequence alone also reduced promoter activity (35.1%). These data indicate that one or more *cis*-regulatory elements needed for *F3H* expression are present in the upstream region 300 bp above the translation start site, and that the *TORE1* insertion definitely affects torenia *F3H* promoter activity *in vivo*.

### Flower color complementation in *F3H*-overexpressed transgenic torenia plants

**Figure 6:**
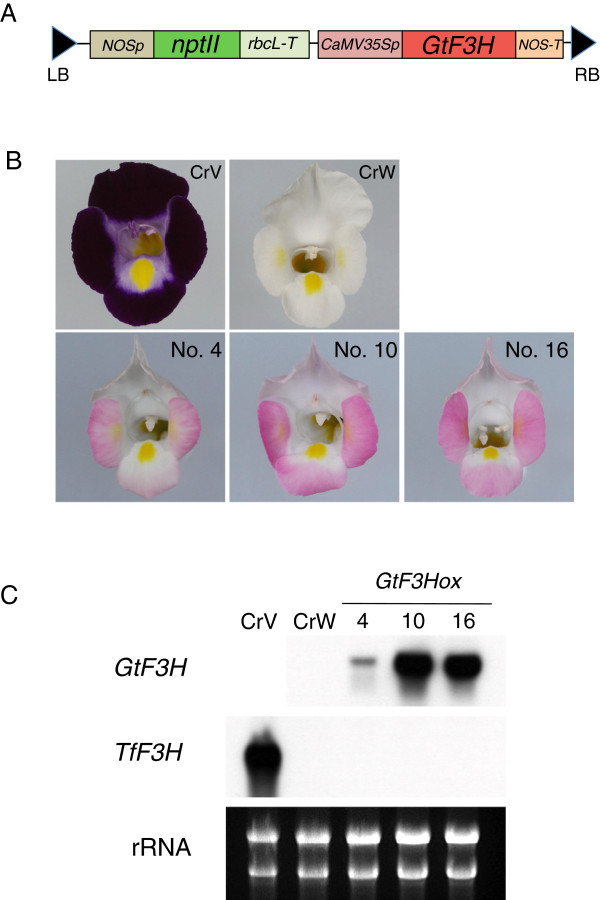
**Complementation of flower color by transformation with gentian *F3H *cDNA.** Stable transgenic plants of CrV were produced via *Agrobacterium*-mediated transformation. **(A)** T-DNA region of binary vector used. **(B)** Flowers of wild-type (CrV and CrW) and *GtF3H*-overexpressing transgenic CrW (nos. 4, 10, and 11) lines. **(C)** Northern blot analysis of petals of transgenic torenia plants. The membrane was probed with foreign gentian *F3H* or endogenous torenia *F3H*. *rRNA* was used as a total RNA loading control.

To confirm whether the deficient *F3H* expression in CrW is actually responsible for the white coloration, CrW was transformed with other functional *F3H* cDNA. To produce transgenic torenia plants, we used a gentian *F3H* gene that encodes a functional F3H enzyme. CrW was transformed with the binary vector pSKan-35SGtF3H (Figure 6A), and 46 transgenic torenia plants were regenerated and cultivated until flowering. Of these transgenic lines, 35 recovered pink-colored pigmentation in flowers, with some variability in the degree of pink color pigmentation; 30 lines had intense pink flowers, and five featured faint pink flowers. The upper lips of transgenic flowers were less pigmented than lower and lateral lips. Representative lines with intense pink (nos. 10 and 16) and faint pink (no. 4) flowers are shown in Figure 6B. Northern blot analysis confirmed the expression of the foreign gentian *F3H* gene, indicating that the transformation was successful in torenia petals. Notably, higher accumulation levels of *GtF3H* transcripts were observed in lines 10 and 16 than in line 4, consistent with recovered flower color intensities (Figure 6C). The endogenous torenia *F3H* gene remained suppressed in CrW and all three transgenic lines. HPLC analysis confirmed that the accumulated anthocyanidin in *GtF3H*-overexpressed CrW was pelargonidin, not delphinidin or cyanidin (Additional file 2: Figure S7), indicating that the B-ring hydroxylation ability of anthocyanins was absent in CrW because of the lack of *F3′H* and *F3′5′H* activities.

## Discussion

In this study, expression analysis of flavonoid biosynthetic genes revealed that only the expression of *F3H* was completely reduced in CrW. F3H is a 2-oxoglutarate-dependent dioxygenase that acts in the flavonoid biosynthetic pathway to hydroxylate flavanones to dihydroflavonols, which are common precursors for major classes of flavonoids, flavonols, catechins, anthocyanins, and proanthocyanidin in plants [1,39]. The gene encoding *F3H* cDNA was first isolated from petunia [40], with homologous genes subsequently cloned in many plant species. *F3H* gene mutations affecting color pigmentation have been reported in various plant species, including petunia [41], soybean [42], Japanese morning glory [24], and carnation [43]. Because anthocyanins are synthesized from dihydroflavonols, reduced *F3H* expression is the likely cause of the torenia CrW colorless flower phenotype.

Analysis of genomic structures did not uncover any mutations in either coding or intron regions of torenia *F3H*, but a long insertion (*TORE1*) was found in the 5′-upstream region of *F3H* in CrW. This insertion was identified as a retrotransposable element of the LTR subtype [44,45]. *TORE1* has typical characteristics of LTR-type retrotransposons, namely 560-bp LTRs with 5 bps of target site duplication (TSD), a PBS, and a PPT, and encodes a partial gag-pol protein (Figure 3; Additional file 2: Figure S3). *TORE1* may thus be a nonautonomous element derived from an originally autonomous one. Southern blot analysis also indicated the presence of *TORE1*-like elements in the torenia genome.

Transient expression analysis using various constructs of the *F3H* promoter revealed that the *TORE1* insertion is indeed involved in reduced promoter activity (Figure 5). For this analysis, we used the heterologous transcription factor genes *GtMYB3* and *GtbHLH1*, which regulate flavonoid biosynthesis in gentian flowers. These two genes were chosen because they are well-characterized and constitute a reliable transient assay system established in our previous studies [33,38]. Although torenia endogenous *TfMYB1* and *TfbHLH1* genes that are probably responsible for regulation of anthocyanin biosynthesis have been recently isolated, the evidence for TfMYB1-TfbHLH1 interaction leading to activation of flavonoid biosynthetic genes remains inconclusive. Further analysis is therefore required to clarify the effects of *TfMYB1* and *TfbHLH1* transcription factor genes on activation of the torenia *F3H* promoter.

Many examples of transposable elements affecting gene expression have been noted in various organisms, with different classes of active transposons observed in genomes of eukaryotes including plants [46,47]. Transposable elements have been confirmed to contribute to plant evolution and diversity [48]. Retrotransposon or retrotransposon-like sequences, in particular, are ubiquitous components of plant genomes, and their impacts on plant genome structure and function have also been revealed (reviewed in [49]). Transposon insertions have been found in all types of genomic sequences, including coding (exon) and noncoding (intron, UTR, upstream, and downstream) regions. In a previous study, an *En*/*Spm*-like transposon (*Ttf1*) insertion in the *TfMYB1* second intron was found to cause a mutant torenia phenotype by reducing expressions of flavonoid biosynthetic genes, including *CHS*, *F3H*, *DFR*, *ANS*, and UDP-glucose: flavonoid glucosyltransferase *(UFGT)*[35]. In our case, the insertion of *TORE1* completely suppressed *F3H* expression, as shown by RT-PCR and northern blot analysis. Transposable element insertions often induce epigenetic changes, such as DNA methylation, that cause strong gene silencing [47,50]; this fact suggests that *TORE1* may reduce *F3H* expression through a similar silencing mechanism. In fact, Southern blot analysis of CrW genomic DNA digested with *Hin*d III revealed some larger bands when probed with *F3H. Hin*d III is sensitive to cytosine methylation within the recognition site, and slowly cleaves hemimethylated AAG^m5^CTT [51]. Partial digestion with *Hin*d III due to the effect of *de novo* methylation has also been reported in transgenic pea plants showing inducible co-suppression of the transgene after virus infection [52]. Such an epigenetic change probably contributes, along with disruption of the *F3H* promoter, to the reduction in *F3H* promoter activity. Further studies using methylation-sensitive restriction enzymes or bisulfite sequencing would confirm this hypothesis.

Detailed examination of the 5′-upstream region of the *F3H* promoter in CrW genomic DNA revealed the presence of a solo-LTR insertion in addition to the full-length *TORE1* insertion (Figure 3; Additional file 2: Figure S4). Southern blotting generated a banding pattern inconsistent with the presence of the solo LTR in the *F3H* promoter, indicating that the *TORE1*-inserted *F3H* and solo LTR-inserted *F3H* are not allelic. The solo LTR is probably derived from a recombination between LTRs within *TORE1* in certain cells. As an example, the formation of solo LTRs through unequal homologous recombination of two LTRs has been previously reported in rice [53]. Based on the presence of solo LTRs and variously truncated fragments in plant genomes, unequal homologous recombination and illegitimate recombination have been inferred to drive genome size decreases in Arabidopsis [54] and rice [55]. CrW sometimes shows faint pink-recovered sections in flower petals (Additional file 2: Figure S8). A *TORE1* excision event is probably not involved in this recovery, however, as class I transposons move by a "copy and paste" process. In the transient expression assay, the solo-LTR insertion was less effective in reducing promoter activity than was the full *TORE1* insertion (Figure 5). This result suggests that the advent of the solo LTR is responsible for the partially recovered phenotype. Further studies are needed to determine the mechanism and developmental timing of this *TORE1* recombination event in the *F3H* promoter of CrW torenia plants.

The white coloration of CrW can clearly be attributed to the *F3H* mutation, a conclusion confirmed by the complementation study (Figure 6). This finding is further supported by the fact that knockdown of torenia *F3H* by RNAi produces white-flowered torenia plants in the blue-flowered cultivar Summerwave Blue [19]. Nevertheless, mutations were also found in *F3′H* and *F3′5′H* gene sequences in CrW (Additional file 2: Figure S2). Deficiency of flavonoid B-ring hydroxylation activities is suggested by two observations: 1) apigenin was the only flavone accumulating in CrW petals (Figure 1) and 2) *F3H*-overexpressing transgenic CrW lines accumulated pelargonidin derivatives, not delphinidin derivatives (Additional file 2: Figure S7). *F3′H* and *F3′5′H* genes in CrW have deletion mutations that should affect enzyme activities. In particular, the single-base deletion in exon 1 of *F3′5′H* results in a frameshift and subsequent premature termination of the enzyme (Additional file 2: Figure S2). Such mutations are frequently observed in other plants. In soybean, for example, single-base deletions in *F3′H* and flavonol synthase *(FLS)* are associated respectively with gray pubescence color [56] and magenta flower color [57]. A 4-bp insertion mutation in UDP-glucose:anthocyanidin 3–*O*-glucoside-2''–*O*-glucosyltransferase (3GGT) induces a dusky flower color in morning glory [58]. In three morning glory species, a single C to T base transition, a single T insertion, and insertion of the transposable element Tip201 in *F3′H* are reportedly involved in reddish flowers [59]. In the case of CrW, the 12-bp deletion in exon 3 of *F3′H* did not cause a frameshift, but apparently resulted in the absence of four amino acid residues (Additional file 2: Figure S2). This deleted amino acid sequence partially overlapped with a threonine-containing binding pocket (A/G-G-X-D/E-T-T/S) regarded as an oxygen-binding motif of cytochrome P450 monooxygenase, required in catalysis [60]. Although *in vitro* enzyme activity was not examined, the lack of this essential motif is very likely responsible for diminished F3′H enzymatic activity. In addition, CrW exhibits a shorter UV maximum absorption peak—333 nm compared with 346 nm in CrV—and recovered anthocyanin pigmentation in *F3H*-overexpressing CrW petals are pink, a color arising from pelargonidin derivatives that lack B-ring hydroxylation (Additional file 2: Figure S7). These results also indicate the absence of F3′H and F3′5′H enzyme activities. It is unlikely, however, that these *F3′H* and *F3′5′H* mutations are responsible for white coloration in CrW. For example, deficiency of hydroxylation activity in the anthocyanidin B-ring caused a color shift, but not to white as reported previously [56,59]. How CrW came to possess *F3′H* and *F3′5′H* mutations is clearly unknown, but they probably arose in this cultivar during breeding selection for flower colors such as pink, lavender, and blue. Another possibility is that the breeding parents of CrW contained the latent mutations of these two genes. Torenia plants of the Crown series have various flower colors, and this variation may depend on genotype. In fact, CrV is heterozygous with respect to these two genes. It is also likely that the other flavonoid biosynthetic genes, besides *F3′H* and *F3′5′H*, have heterozygous mutations, because torenia is primarily an outcrossing species. Further analysis of other cultivars of the Crown series should also provide clues useful for answering this question.

## Conclusions

In summary, we investigated the causal factor(s) of white flower color in Crown White, a torenia cultivar. Using transient and transgenic approaches, we demonstrated that a novel retrotransposable element (*TORE1*) inserted into the 5′-upstream region of the *F3H* gene is directly responsible for the colorless flower phenotype. Further investigations will be required to understand the dynamics of *TORE1* in the torenia genome. As torenia cultivars have been produced in various flower colors, we expect that additional mutations will soon be uncovered using molecular approaches. We believe that the Crown White cultivar will be useful for genetic engineering of novel flower colors using various plant pigments.

## Methods

### Plant materials

*Torenia fournieri* plants were used in this study. White-flowered Crown White (CrW) and violet-flowered Crown Violet (CrV) cultivars selected from ‘Crown Mix’ (PanAmerican Seed Company, West Chicago, IL, USA) were kindly provided by Dr. Ryutaro Aida (National Institute of Floricultural Science, Japan). The plants were cultivated in a greenhouse under natural conditions from spring to summer. For transformation experiments, *in vitro* cultured plants were used as described previously [61].

### Analysis of pigment components in torenia flower petals

Fully opened flower petals of CrV and CrW were used. Flower petals were extracted in 0.1% HCl-methanol with gentle shaking overnight at 4°C. Spectrophotometric analysis was performed using a SpectroMax 190 absorbance microplate reader (Molecular Devices, Sunnyvale, CA, USA). Flavonoid aglycones of anthocyanins and flavones in flower petals were analyzed by high-performance liquid chromatography (HPLC) as described previously [62].

### Expression analyses of flavonoid biosynthetic genes

Total RNA was isolated from opening flowers (stage 4 as defined by Ueyama et al. [9]) using a FastRNA pro GREEN kit (Qbiogene, Irvine, CA, USA). cDNAs were synthesized from total RNA after removal of genomic DNA using a PrimerScript RT reagent kit with gDNA Eraser (Takara Bio, Shiga, Japan). Semi-quantitative RT-PCR analysis was performed using primer sets listed in Additional file 1: Table S1 for nine flavonoid biosynthetic genes from *T. hybrida*: chalcone synthase (*CHS,* accession no. AB012923), chalcone isomerase (*CHI,* AB548584), flavanone 3-hydroxylase (*F3H,* AB211958), dihydroflavonol 4-reductase (*DFR,* AB012924), anthocyanidin synthase (*ANS,* AB044091), flavonoid 3*′*-hydroxylase (*F3′H,* AB057672), flavonoid 3*′*,5*′*-hydroxylase (*F3′,5′H,* AB012925), anthocyanin 5-*O*-glucosyltransferase (*5GT,* AB076698)*,* and flavone synthase II (*FNSII,* AB028152). An actin gene (*ACT3,* AB330989) was used as an endogenous control. Each 50-μL reaction mixture contained 1× Ex *Taq* buffer, 200 μM dNTPs, 0.5 μM of each primer, 5 units Ex *Taq* polymerase (Takara Bio), and 1 μL cDNA template. PCR conditions were as follows: 1 min 30 s at 94°C, followed by 26 to 34 cycles of 20 s at 94°C, 40 s at 55°C, and 2 min at 72°C, and a final extension of 10 min at 72°C. The PCR products were separated by electrophoresis on a 1.0% agarose gel in TAE buffer and stained with ethidium bromide.

Total RNAs (5 μg) were subjected to northern blot analysis. Probes for torenia *F3H*, *F3′H*, and *F3′5′H* were prepared using a PCR-DIG Probe synthesis kit (Roche Diagnostics, Basel, Switzerland) using primers pairs listed in Additional file 1: Table S1. Hybridization and detection were performed using a DIG Nucleic Acid Detection kit (Roche Diagnostics).

### Determination of torenia *F3H*, *F3′H*, and *F3′5′H* gene sequences

Genomic DNAs were isolated from leaves of each cultivar using a Nucleon PhytoPure kit (GE Healthcare, Little Chalfont, UK). PCR reactions were performed as described above, except that genomic DNAs were substituted for the cDNA templates. The primer pairs used are listed in Additional file 1: Table S1. The amplified fragments were subcloned into a pCR4TOPO TA cloning vector (Life Technologies, Carlsbad, CA, USA) and sequenced using a BigDye Terminator version 1.1 cycle sequencing kit on an ABI 3130 genetic analyzer (Life Technologies).

### Isolation of 5′-upstream regions of the torenia *F3H* gene

The 5′-upstream region of the torenia *F3H* gene was identified using inverse PCR. One microgram of genomic DNA of CrV was digested with the restriction enzyme *Hin*d III and self-ligated using a Takara ligation kit version 3.0 (Takara Bio). Inverse PCR was performed in 25-μl reaction mixtures containing 100 ng ligated genomic DNA, 1× *LA* buffer, 2.5 mM MgCl_2_, 400 μM dNTPs, 0.2 μM of each primer, and 1.25 units of *LA Taq* polymerase (Takara Bio). The primer sets used are described in Additional file 1: Table S1. Reaction conditions consisted of pre-heating at 94°C for 90 s, 35 cycles at 95°C for 20 s, 60°C for 40 s, and 72°C for 3 min, and an extension at 72°C for 10 min. Amplified fragments of about 5 kb for *F3H* were subcloned and sequenced as described above. The putative transcriptional initiation site was determined by 5′*-*RACE technology using a GeneRacer kit (Life Technologies). Several primers designed from the sequence of the 5′-upstream region of CrV were used for PCR amplification of CrW. The PCR products were also subcloned and sequenced as described above.

### Southern blot analysis of torenia *F3H*, *F3′H*, and *F3′5′H* genes and *TORE1*

Genomic DNAs (10 μg) were digested with *Hin*d III, *Eco *RI, or *Xba* I, followed by separation on 1% agarose gels and transfer to nylon membranes. Coding sequences of *F3H*, *F3′H*, and *F3′5′H* were used as probes. LTR and gag-pol protein sequences were also used. Hybridization and detection was performed as described previously [34].

### Transient expression assays of *F3H* promoter activities using Arabidopsis suspension cells

*Arabidopsis thaliana* suspension cell line T87 was provided by RIKEN BRC, a participant in the National Bio-Resource Project of the MEXT (Ministry of Education, Culture, Sports, Science and Technology), Japan. Protoplast isolation and transfection experiments for transient expression assays were performed as described by Hartmann et al. [63]. 35Spro-GtMYB3 and 35Spro-GtbHLH1 vectors were used as effector vectors [33]. An approximately 1-kb long 5′-upstream region prior to the translation start site of the torenia *F3H* gene was amplified and used as the *TfF3H* promoter. *TfF3H* promoters with or without transposon insertions were fused to *LUC* (firefly luciferase gene). The gentian *GtF3H* promoter was also used. 35S-RLUC (*Renilla* luciferase gene) was coinfected as a transformation control. Dual luciferase assays were performed as described previously [64].

### Complementation study by transformation of torenia plants

A binary vector was constructed to express a gentian *F3H* cDNA (*GtF3H-1*[62], accession no. AB193311) in CrW. The plasmid, pSKan-35SGtF3H, was transformed into *Agrobacterium tumefaciens* strain EHA101. Torenia transformation was performed as described previously [65] using kanamycin as a selection agent. The generated transgenic plants were transferred to an enclosed greenhouse and cultivated until flowering. Northern blot analysis was performed as described above using probes for torenia and gentian *F3H*.

## Abbreviations

F3H: Flavanone 3-hydroxylase; F3′H: Flavonoid 3*′*-hydroxylase; F3′5′H: Flavonoid 3*′*,5*′*-hydroxylase; LTR: Long terminal repeat; TORE1: Torenia retrotransposon 1.

## Competing interests

The authors declare that they have no competing interests.

## Authors’ contributions

MN conceived and designed the experiments. EY, MS, and KF carried out the experiments. MN and TN also performed some of the experiments. HT helped to analyze pigments. MN supervised the study and wrote the manuscript. HT and TN critically revised the manuscript and completed it. All authors read and approved the final manuscript.

## Supplementary Material

Additional file 1: Table S1 List of primer sequences used in this study. Click here for file

Additional file 2: Figure S1 Expression analyses of two transcription factor genes in CrV and CrW. RT-PCR analysis of *TfMYB1* and *TfbHLH1* and the β-actin gene. **Figure S2.** Schematic structure and mutated sequences of *ThF3′H* and *ThF3′5′H* genes. (A) The *ThF3′H* gene consists of three exons and two introns. (B) The *ThF3′5′H* gene consists of two exons and one intron. **Figure S3.** Sequence of the 5′-upstream region of *F3H* in CrW. **Figure S4.** Insertion of the solo-LTR in 5′-upstream region of *F3H* in CrW. **Figure S5.** Southern blot analysis of *F3′H* and *F3′5′H* in CrV and CrW. **Figure S6.** Schematic diagram of the genomic structure of the *F3H* gene in CrV and CrW. **Figure S7.** HPLC analysis of flower petal anthocyanidins in *GtF3H*-overexpressing transgenic CrW. **Figure S8.** Example of pigment recovery in a CrW petal. (A) Whole flower. (B) Magnification of boxed red area in A. Click here for file
