# Supplementary material for: Molecular characterization of mutations in white-flowered torenia plants
Source: BMC Plant Biol. 2014 Apr 2;14:86. doi: 10.1186/1471-2229-14-86 (PMC4234012; doi:10.1186/1471-2229-14-86)
Supplement: Additional file 1: Table S1 — List of primer sequences used in this study. [file 1471-2229-14-86-S1.pdf]

Supplementary Table S1. Primers used in this study.

Primer		Sequence	Expected fragment length
**RT PCR analysis**			
CHS	Forward	5′-GATTCGMAGGGCTCAGAGAGCTGAAGG-3′	1143bp
	Reverse	5′-GGGCGTGCTGTGMAGSACAACGGTCTC-3′	
CHI	Forward	5′-AAGAACGAAGTATTCCCACCAG -3′	335bp
	Reverse	5′-TCCACGATCATTGACCTAAACC-3′	
F3H	Forward	5′-ATGGCACGAGCAGGACCACTAACCCTA -3′	1104bp
	Reverse	5′-CTATTTAGTTTTCCCATTGTTAAGATG -3′	
DFR	Forward	5′-GTATGAGCATGGAAGTAGTAGTACCAAAAG -3′	1060bp
	Reverse	5′-TGCATGTTCTATTCTATCTTATGTTCTCCA -3′	
ANS	Forward	5′-TCCCAAAGAACATGGTTTCTCCAGCATCTC-3′	1172bp
	Reverse	5′-CCAAAACAATCATCAAACAATAAACTCCAC-3′	
F3'H	Forward	5′-CCATGAGTCCCTTAGCCTTGATGATCCTAA-3′	1632bp
	Reverse	5′-CCATACCATACCATACCATGCAAACTCACA-3′	
F3'5'H	Forward	5′-GTACTCCGTCCGACCTTTTATTGTATGG-3′	1617bp
	Reverse	5′-GGATTGGATCTATGGAAGATAACAATGT-3′	
5GT	Forward	5′-AATGGTTAACAAACGCCATATTCTACTAGC-3′	1477bp
	Reverse	5′-CGACGTCCATAACAACAATAATAACATATA-3′	
FNSII	Forward	5′-ATGGACACAGTCTTAATCACACTCTACACC-3′	1549bp
	Reverse	5′-CCCTACTACATCAAGCACCCGATATTGTGG-3′	
ACT3	Forward	5′-GCTGTTCTCTCCCTTTATGC-3′	921bp
	Reverse	5′-GCACACAGAGAATAGCAAAC-3′	
MYB1	Forward	5′-TTCGCGTTCAAATGAGTAGAGACAAAATAT-3′	880bp
	Reverse	5′-ATAATTAATTACCACCATAACATGGTCTCC-3′	
bHLH1	Forward	5′-ATGGACAATGAAGTGGTTACAGAGAATCTA-3′	1905bp
	Reverse	5′-TCAACTCTTCTTGATAACTTTCTGAAGAGCC-3′	
**Amplification of genomic sequences**			
F3H	Forward	Same as RT-PCR	
	Reverse	Same as RT-PCR	
F3'H	Forward	5′-ATTAGCCATGAGTCCCTTAGCCTTGATG-3′	
	Reverse	5′-TACAAGCACCATACCATACCATACCATG-3′	
F3'5'H	Forward	Same as RT-PCR	
	Reverse	Same as RT-PCR	
**Amplification of 5′upstream regions of *F3H***			
Inverse PCR	Forward	5′-TCTCTGGGATTGACGATGACGACGATGAAT -3′	
	Reverse	5′-AAACTTTTCATGCAGCGATTTTTCGAGCGC -3′	
Ttransient expression assay			
for 1k, 1.6k, 4.5kbp promoters	Forward	5′-CCTGCAGGAGTTGGGCAAACGCTACCA-3′	
	Reverse	5′-ACTAGTTGGTCCTGATCGCGCCATATG-3′	
for 300bp promoter	Forward	5′-CCTGCAGGTGTATTCCAAAATATCAAT-3′	
	Reverse	5′-ACTAGTTGGTCCTGATCGCGCCATATG-3′	
**Probes for northern blot and southern blot analysis**			
F3H	Forward	Same as RT-PCR	
	Reverse	Same as RT-PCR	
F3'H	Forward	Same as RT-PCR	
	Reverse	Same as RT-PCR	
F3'5'H	Forward	Same as RT-PCR	
	Reverse	Same as RT-PCR	
LTR of Tft1	Forward	5′-TTGTTGAGAATTCTTGCTCT-3′	
	Reverse	5′-TGTGGAGAAATTAGCTCATG-3′	
Gag-pol protein of TORE1	Forward	5′-ATGGCTGCGAGGTTCGAAGT -3′	
	Reverse	5′-CTACTTGCCCACTGTTGTTC -3′	
