# Supplementary material for: Molecular characterization of mutations in white-flowered torenia plants
Source: BMC Plant Biol. 2014 Apr 2;14:86. doi: 10.1186/1471-2229-14-86 (PMC4234012; doi:10.1186/1471-2229-14-86)
Supplement: Additional file 2: Figure S1 — Expression analyses of two transcription factor genes in CrV and CrW. RT-PCR analysis of TfMYB1 and TfbHLH1 and the β-actin gene. Figure S2. Schematic structure and mutated sequences of ThF3′H and ThF3′5′H genes. (A) The ThF3′H gene consists of three exons and two introns. (B) The ThF3′5′H gene consists of two exons and one intron. Figure S3. Sequence of the 5′-upstream region of F3H in CrW. Figure S4. Insertion of the solo-LTR in 5′-upstream region of F3H in CrW. Figure S5. Southern blot analysis of F3′H and F3′5′H in CrV and CrW. Figure S6. Schematic diagram of the genomic structure of the F3H gene in CrV and CrW. Figure S7. HPLC analysis of flower petal anthocyanidins in GtF3H-overexpressing transgenic CrW. Figure S8. Example of pigment recovery in a CrW petal. (A) Whole flower. (B) Magnification of boxed red area in A. [file 1471-2229-14-86-S2.pdf]

Additional file 2: Figure S1

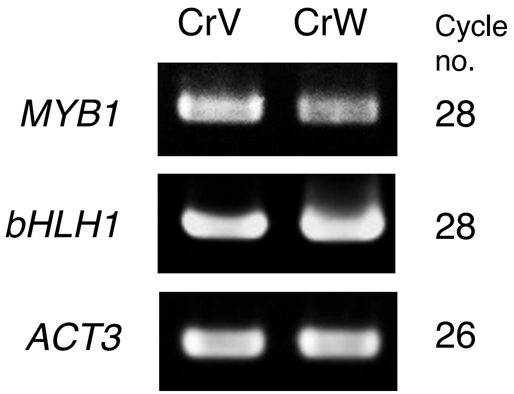

Fig. S1 Expression analyses of two transcription factor genes in CrV and CrW.

RT-PCR analysis of *TfMYB1* and *TfbHLH1* and the β-actin gene. *TfMYB1* and *TfbHLH1* are suggested to be involved in anthocyanin biosynthesis in torenia. Gene names and PCR cycles are shown to the left and right of each panel, respectively.

Additional file 2: Figure S2

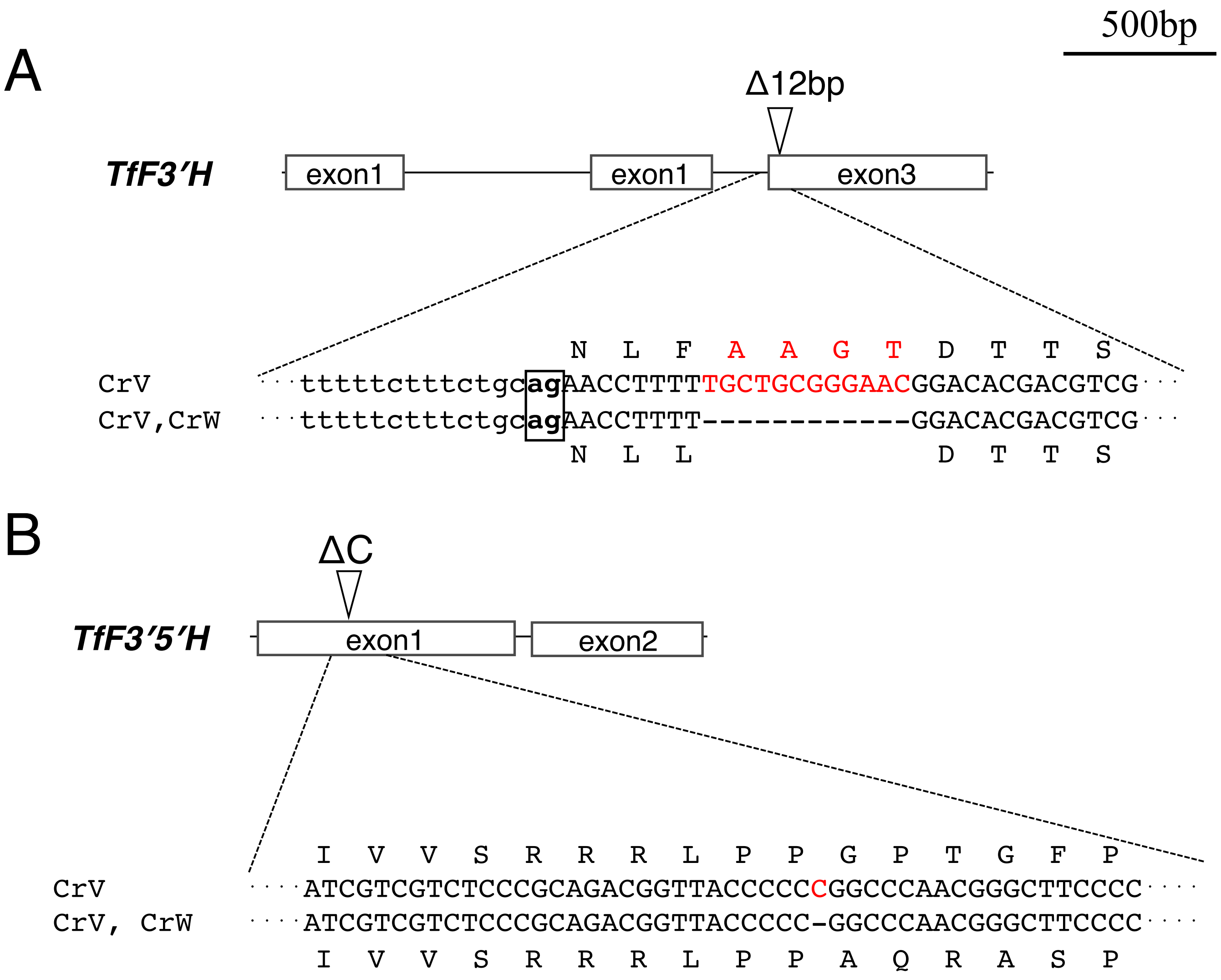

Fig. S2 Schematic structure and mutated sequences of *ThF3'H* and *ThF3'5'H* genes.

(A) The *ThF3'H* gene consists of three exons and two introns. The deduced amino acid sequence corresponding to the 12-bp deletion in CrW is shown. Small letters indicate intron sequence, and an acceptor site (ag) is boxed. (B) The *ThF3'5'H* gene consists of two exons and one intron. The deduced amino acid sequence corresponding to the cytosine deletion in CrW is shown.

Additional file 2: Figure S3

```
...GCACGAACCAAGTCATTCATTTAATGATGTGAGAAAATGTCATACTTTAATTGATATTTTGGAATACATGTGGAGAAATTAGCTCATGAACTTGGCTAGTTGGAGCTGAAA
                                                                    TSD
ACTGGTTGCATGGTTTTGTTTCTGGTTGTGTGAAGAATCGTCGATGCTTATGAGAAGAAAATGCAGTGGAAGAGTAAAGAGTTATAAGTGTGTTATCACTTATAAAGTTGC
TGTTTACTATATCACGTTTTCTTTGAGAGCTGCACATGGGAACGGTTAATGAGCAGTTATTAAGCTGAAGCAAGATTGTGTGTTCTGTTAGTTCGGTTTCAAGTAGCAGTT
TTGCAGATCTCATAACTTGGTGTGTAGTTAGCCAATTTAAGCGATTTAAATTGCGTTGAAAAGCTCACATTACATACAACACATTGTAACTCATCATACTCAATAAAATTC
TCTCTTCGTCCCGTGGAGTAGGGCTAAAGTTAGCGCCGAACCACGTTAAATCTGTTTTCTTCTGAGACTTTTCTGATTTTCTGTTCTTATTCAATCAAGTTTCTTTCGATC
CAAACGCGTAGTAAACATATCACAAGACTTGTTAACTTTAATCTTGAGCATCTAAGAGCAAGAATTCTCAACAAGTGGTGCGGTGAGCGTGGATAACTCGAAGATGGCTGC
                                                                            PBS                M  A  A
GAGGTTCGAAGTTGAAAAGTTCACGGGTGATAATGACTTCGGTTTGTGGAAAATGAAGATGAGGGCGTTGCTTACCCAACAAGGGCTGATAGAGGTGTTGATGGTGGAGGA
R  F  E  V  E  K  F  T  G  D  N  D  F  G  L  W  K  M  K  M  R  A  L  L  T  Q  Q  G  L  I  E  V  L  M  V  E  D
TCCACCGGCCACAGTGGTGGCTGGGACTGCTCCGACGGGCCAAGAAGATGCCGCTGCTGCAGCTGTAAATGCACAAGCTGCGGCAGAGAAGAAAATCTTGGATTCGAAGGC
P  P  A  T  V  V  A  G  T  A  P  T  G  Q  E  D  A  A  A  A  A  V  N  A  Q  A  A  A  E  K  K  I  L  D  S  K  A
GCATTCAGTGATCATTCTGAGTTTGGGAGATCGAGTCTTGCGTCAAGTCTCTCATGAATCAACCGCTCTTGGTCTGTGGAAGAAATTGGAAGAGCTTTACATGACAAAGTC
H  S  V  I  I  L  S  L  G  D  R  V  L  R  Q  V  S  H  E  S  T  A  L  G  L  W  K  K  L  E  E  L  Y  M  T  K  S
TCTTGCCAATCGACTTTATCTCAAGCAAGCGCTCTATTCGTTCAAGATGATTGAAGAAAAGGCCATTGATGAGCAGATGGATCAGTTTATCAAGCTGATTTTGGATCTTGA
L  A  N  R  L  Y  L  K  Q  A  L  Y  S  F  K  M  I  E  E  K  A  I  D  E  Q  M  D  Q  F  I  K  L  I  L  D  L  E
AAACATCGAAGTGAAGATAGAGGATGAGGATCAAGCCTTGCTCTTGGTGTGTGCTTTGCCTCGGAGTTACAACACATTCAAGGACACGTTGTTGTATGGCCGAGAGACTTT
N  I  E  V  K  I  E  D  E  D  Q  A  L  L  L  V  C  A  L  P  R  S  Y  N  T  F  K  D  T  L  L  Y  G  R  E  T  L
GACTTTGAAAGAGGTGCAAGCCGCGTTGAAGTCGAAACAGCTGAACACGAGGATTGATAACAAGGCAGTGGGATCTACATCCGAGGCTCTTTATGTTAAAGGGAAAGGTGA
T  L  K  E  V  Q  A  A  L  K  S  K  Q  L  N  T  R  I  D  N  K  A  V  G  S  T  S  E  A  L  Y  V  K  G  K  G  E
AGAGAAGAAAACACACAAAGAAAGAAAGAACAAGTCCAAAAAGAAGGTTAAATGTTTCTATTGCGATGAGGAGGGTCATATGTGCAAGAATTGTCCCAAGAAGGAGAGAGA
E  K  K  T  H  K  E  R  K  N  K  S  K  K  K  V  K  C  F  Y  C  D  E  E  G  H  M  C  K  N  C  P  K  K  E  R  D
CAAGGGCAAGAAAGTGGAGCAAGGCGAGGCTGCAATGGCTTGTGAAAGTTATGAGTCGGCTGACGTCTTGGCCGTGACTCATGAAGATCAAGATGTGACCAAATCTGAAAA
K  G  K  K  V  E  Q  G  E  A  A  M  A  C  E  S  Y  E  S  A  D  V  L  A  V  T  H  E  D  Q  D  V  T  K  S  E  K
ATCTGGTAAATGGCTACTTGATTCCGCTTCTTCTTTTCATGTCACATGTGTTAAGTCTTGGATTAAAGACTTTAAGGGTTGTGATGGTTGTCTGGTCTCTGTTGGGGAAGA
S  G  K  W  L  L  D  S  A  S  S  F  H  V  T  C  V  K  S  W  I  K  D  F  K  G  C  D  G  C  L  V  S  V  G  E  E
AAAGCAATACAAAATTCTGGGCTTTGGAACGGTTAAAATAAGGCTTAAAACCGGTGGAGTAAGAATTTTGAGAAATGTGAAGTTCATTCCGGATTTGGGTAGAAACCTTAT
K  Q  Y  K  I  L  G  F  G  T  V  K  I  R  L  K  T  G  G  V  R  I  L  R  N  V  K  F  I  P  D  L  G  R  N  L  I
TTCTGTAGGGTTGTTGGATGTTCAAGGCTTTAAGTGTGTTGCTGGAAATGGTGTGATGAAGGTTTTCAAAGGGTCTAAGGTGATTATGAGTGGTACTTTGCAGAAAAACAG
S  V  G  L  L  D  V  Q  G  F  K  C  V  A  G  N  G  V  M  K  V  F  K  G  S  K  V  I  M  S  G  T  L  Q  K  N  R
AACTTATCATGTTACAGGATCTGAAACAGTGGTGAACTGGGCTGGTTTGGGTGCTCGTAAAATCTGCTGTGGTGATTCTGGGAGTGAGAGGTTTGTTCAGGGTAATAGTAT
T  Y  H  V  T  G  S  E  T  V  V  N  W  A  G  L  G  A  R  K  I  C  C  G  D  S  G  S  E  R  F  V  Q  G  N  S  I
CCTATGTGGTGATCATGATAAGGCCGGGTTTGAGCAGGTTACACAAGGGGAGATCACAGATCAGAGGGCTCAAAGTGAGGGATCTGACACTTTATTGTCTGAGGTGGAGCA
L  C  G  D  H  D  K  A  G  F  E  Q  V  T  Q  G  E  I  T  D  Q  R  A  Q  S  E  G  S  D  T  L  L  S  E  V  E  Q
GATTTGTGTGAGAGTTGGTAGAGTTCTTTGTCCAGTTTCTGGGAAAGATTTGAATTATCTGAGGAATGGGGATATTCCTGAGGCTGATTGTGGTGGCCAACTACAAGGATC
I  C  V  R  V  G  R  V  L  C  P  V  S  G  K  D  L  N  Y  L  R  N  G  D  I  P  E  A  D  C  G  G  Q  L  Q  G  S
GAATCTGCAAGTGACAAAGGAAGTGGGACTTATCAAGTTTGAAAGGCTGAAAAATGTGATTTCTGAGTCGTGTTTGGTGGTGGATAACTGTGGTGCTTCGAGTTTTCCTGG
N  L  Q  V  T  K  E  V  G  L  I  K  F  E  R  L  K  N  V  I  S  E  S  C  L  V  V  D  N  C  G  A  S  S  F  P  G
GGCTGCTGAAGATCAAACTCAGTTTAATCTGCAGAATGGGAGTTGGTTTACAGCTGAGGTGGAGGAAAATGAAGCAAATGGTCAGTTTTATGAATGCTCAAAATCTGATGA
A  A  E  D  Q  T  Q  F  N  L  Q  N  G  S  W  F  T  A  E  V  E  E  N  E  A  N  G  Q  F  Y  E  C  S  K  S  D  D
TGGCAGCAAGCTTGGAACAACAGTGGGCAAGTAGGTACCTTTGCGAGGTTGTAGCAGGCTGTGTGGGTGGATTTTGAGGAGGCTGTGGGATGCTTCGGGTTCACATCATAT
G  S  K  L  G  T  T  V  G  K  *
GGTACTTCAATGTTCTGGGGATTTAACTCAGGTGGAGTTAAAGAGGAATCCACAAATTAAGCTCAAAGTACTCGGTTTGCGCATATTTGGTACAGAGTTTCTGGTTTGTTT

TTGGGGGCTAAAACTTGGTTTTCTGGGCTGTTAACAAGTTGTTCTTGTGCTGAGAAAGGTTGTTAGCTGTGAGGTAACCAGAGTTGTGGATGTAGAACTGAAGTTTGGACT
TGATGTGATAGTATTTGAGGTTGATCGAGTGAATACAAGTTCGCTGAAAAATTCTCAAATTTGTGTTCGTTTGGCTGGTTTCTGGGGCGTGTTCTGGATCGTTCTTGAGGG
AATTCAGCTGGATGTTGTTTGCTTGGGAAAGTAGGCATGGTGGACTGAAGAGGGAAGTGGAGAGTTAATGAGCTAAGGTGGAGATTTGTGGAGAAATTAGCTCATGAACTT
                                                PPT
GGCTAGTTGGAGCTGAAAACTGGTTGCATGGTTTTGTTTCTGGTTGTGTGAAGAATCGTCGATGCTTATGAGAAGAAAATGCAGTGGAAGAGTAAAGAGTTATAAGTGTGT
TATCACTTATAAAGTTGCTGTTTACTATATCACGTTTTCTTTGAGAGCTGCACATGGGAACGGTTAATGAGCAGTTATTAAGCTGAAGCAAGATTGTGTGTTCTGTTAGTT
CGGTTTCAAGTAGCAGTTTTGCAGATCTCATAACTTGGTGTGTAGTTAGCCAATTTAAGCGATTTAAATTGCGTTGAAAAGCTCACATTACATACAACACATTGTAACTCA
TCATACTCAATAAAATTCTCTCTTCGTCCCGTGGAGTAGGGCTAAAGTTAGCGCCGAACCACGTTAAATCTGTTTTCTTCTGAGACTTTTCTGATTTTCTGTTCTTATTCA
ATCAAGTTTCTTTCGATCCAAACGCGTAGTAAACATATCACAAGACTTGTTAACTTTAATCTTGAGCATCTAAGAGCAAGAATTCTCAACAATACAATTAATAGGTACTCT...
                                                                                                      TSD
```

Fig. S3 Sequence of the 5′-upstream region of *F3H* in CrW. The figure shows 5 bp of the target site duplication (TSD; blue letters), 560 bp of long terminal repeats (LTRs; red letters), the primer binding site (PBS; green letters), and the polypurine tract (PPT; brown letters). The amino acid sequence deduced from the open reading frame (ORF) is indicated by black letters.

Additional file 2: Figure S4

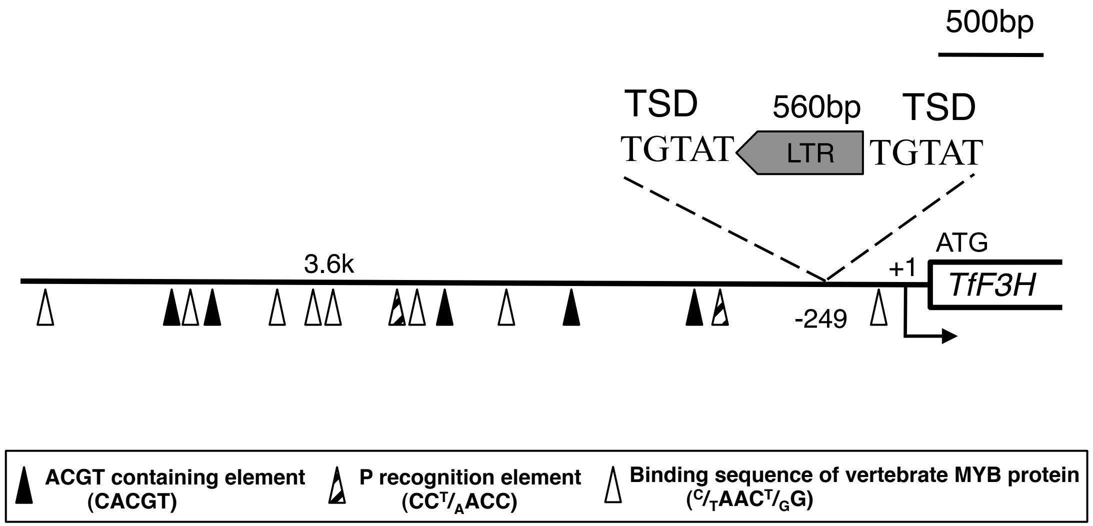

Fig. S4 Insertion of the solo-LTR in 5′-upstream region of *F3H* in CrW.

A putative solo-LTR of 560 bp was identified in *F3H* promoter of CrW. It was flanked by 5-bp target site duplications (TSD) and the position was identical to the *TORE1* insertion site.

Additional file 2: Figure S5

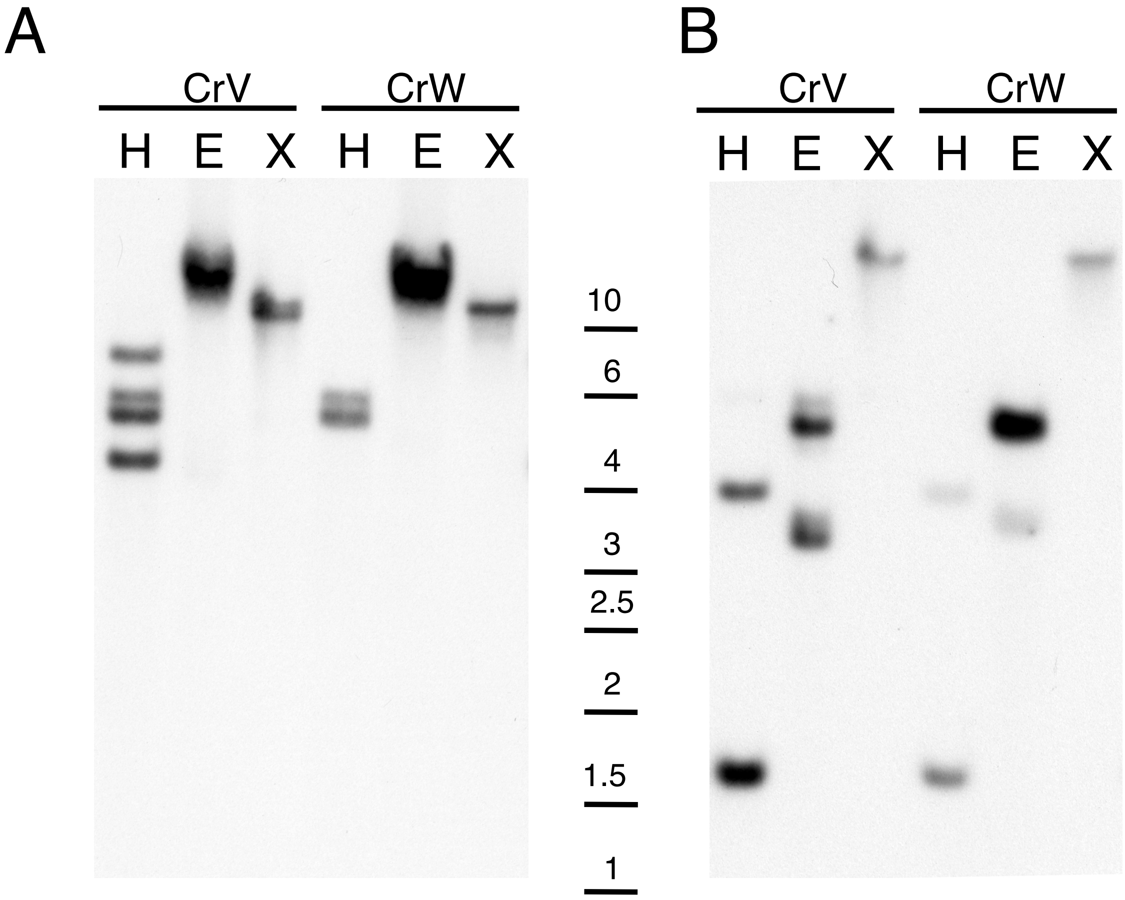

Fig. S5 Southern blot analysis of *F3'H* and *F3'5'H* in CrV and CrW.

Total genomic DNAs were digested with *Hin*d III (H), *Eco* RI (E), and *Xba* I (X) and transferred to nylon membranes as described in Methods. Membranes were probed with DIG-labeled sequences of *F3'H* (A) and *F3'5'H* (B). DNA marker sizes (kbp) are shown.

Additional file 2: Figure S6

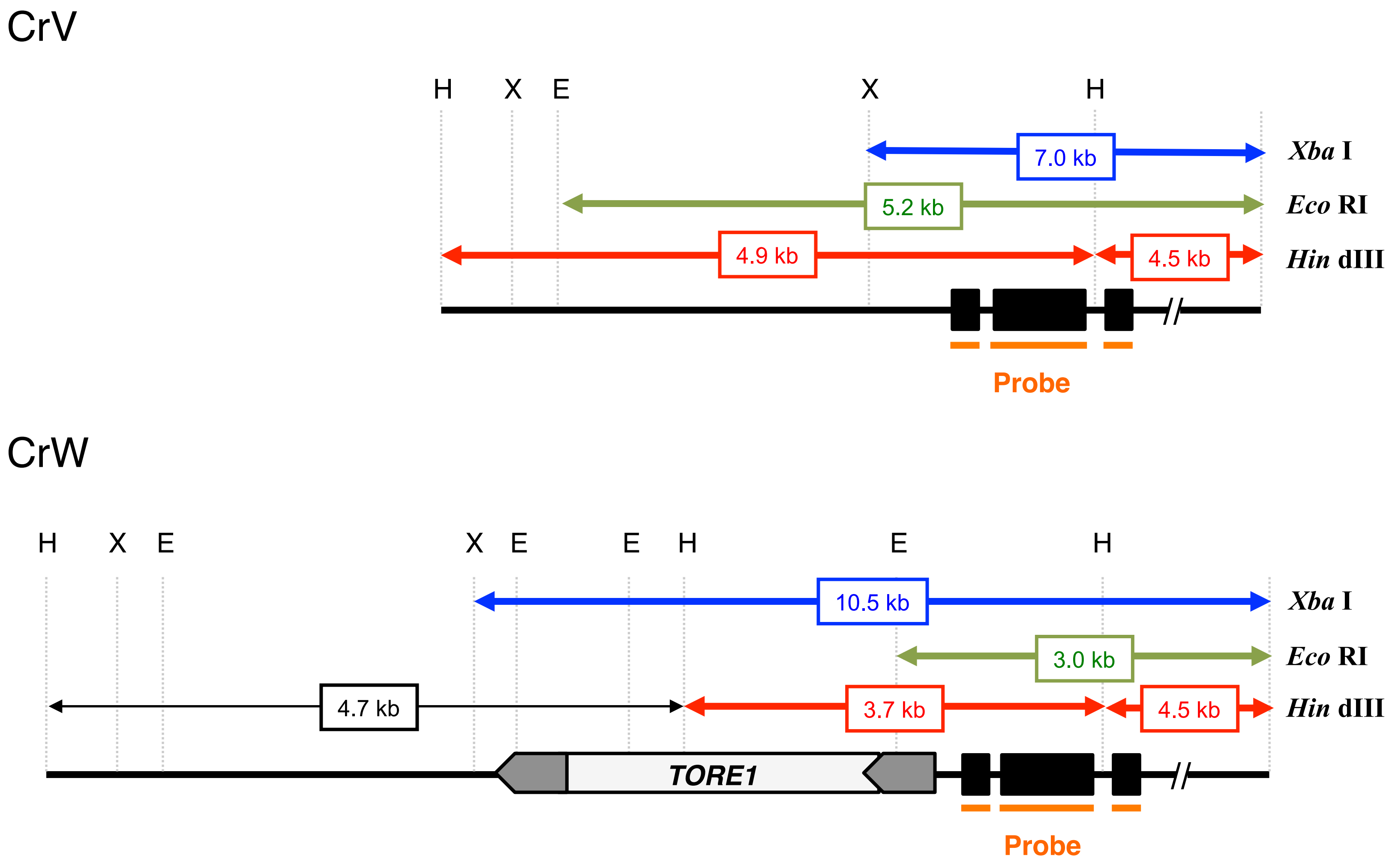

Fig. S6 Schematic diagram of the genomic structure of the *F3H* gene in CrV and CrW.

Sites of restriction enzymes *Xba* I, *Eco* RI, and *Hin*d III used in the Southern blot analysis are shown. Restriction sites at the 3′-proximal region were deduced from the results of Figure 4. Lengths following complete digestion are shown in blue, green, and red for *Xba* I, *Eco* RI and *Hin*d III, respectively. The *F3H* probe used is shown in orange.

Additional file 2: Figure S7

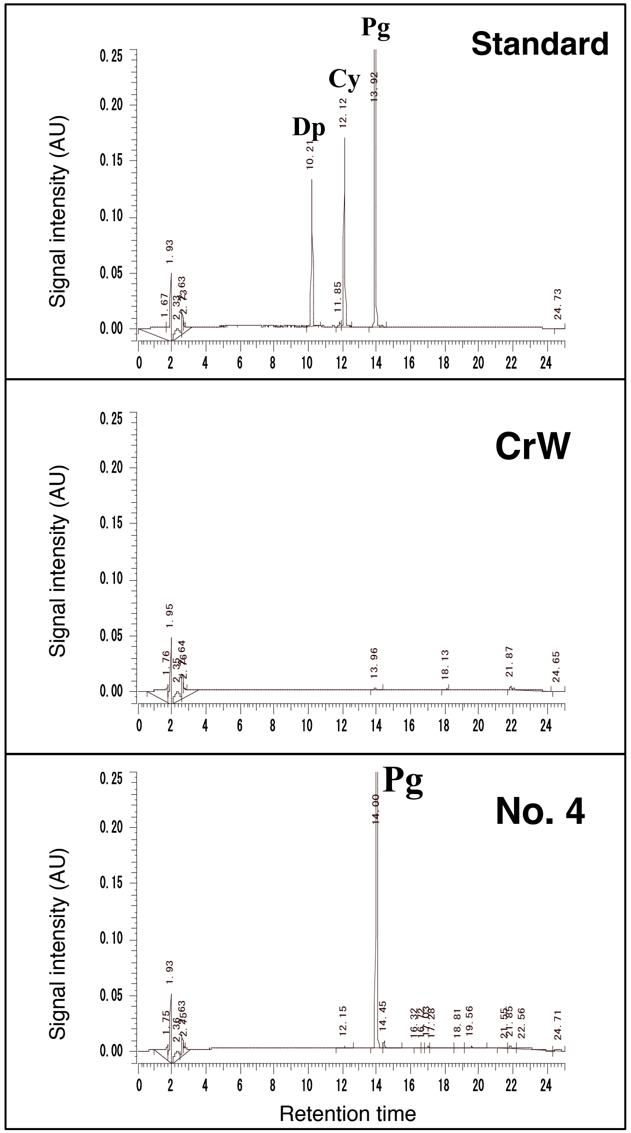

Fig. S7 HPLC analysis of flower petal anthocyanidins in *GtF3H*-overexpressing transgenic CrW.

After acid hydrolysis of petal extracts, the hydrolysates containing anthocyanidins were subjected to HPLC analysis.
Dp, Cy, and Pg correspond to delphinidin, cyanidin, and pelargonidin, respectively.

Additional file 2: Figure S8

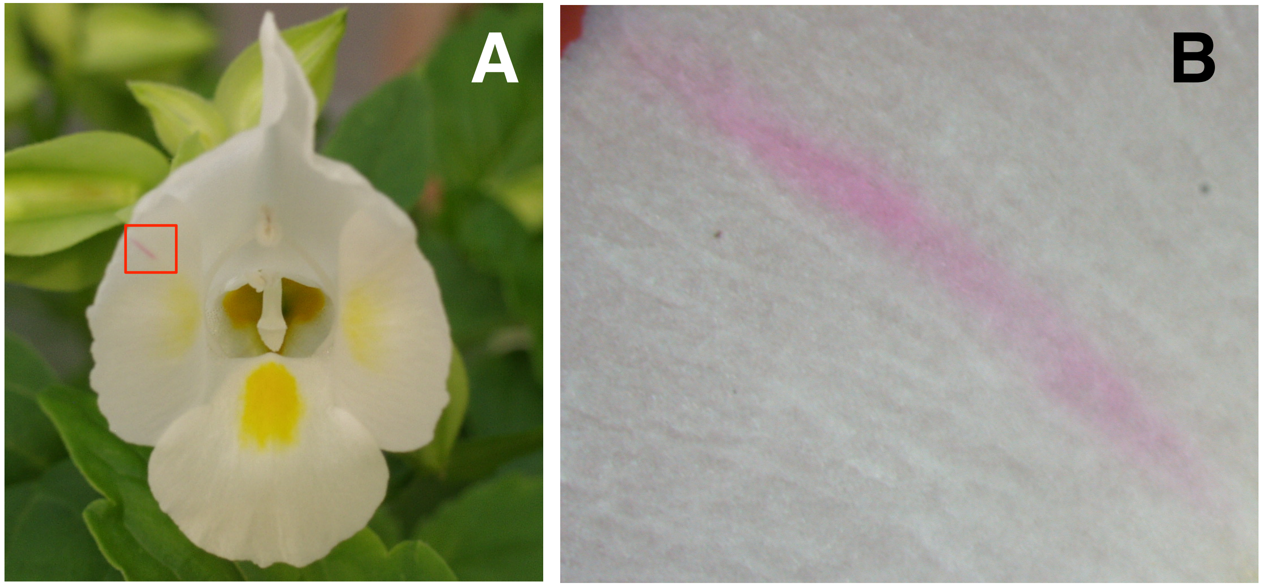

Fig. S8 Example of pigment recovery in a CrW petal.

(A) Whole flower. (B) Magnification of boxed red area in A.
